# Promoting a growth mindset decreases behavioral self-handicapping among students who are on the fixed side of the mindset continuum

**DOI:** 10.1038/s41598-022-11547-4

**Published:** 2022-05-06

**Authors:** Lilla Török, Zsolt Péter Szabó, Gábor Orosz

**Affiliations:** 1 ULR 7369-URePSSS-Unité de Recherche Pluridisciplinaire Sport Santé Société, Sherpas, Univ. Lille, Univ. Artois, Univ. Littoral Côte d’Opale, Liévin, France; 2 Department of Psychology and Sport Psychology, University of Physical Education, Budapest, Hungary; 3 Department of Ergonomics and Psychology, Budapest University of Technology and Economics, Budapest, Hungary; 4 Department of Social and Organizational Psychology, University of Pécs, Pécs, Hungary

**Keywords:** Psychology, Human behaviour

## Abstract

The present study examined the interplay between chronic intelligence beliefs, manipulated intelligence beliefs and self-handicapping processes. Prior studies showed that holding more of a fixed intelligence theory makes one vulnerable to resorting to self-protective mechanisms such as self-handicapping, while growth intelligence mindset can serve as a protective factor for self-handicapping. However, no prior studies have examined the potential interaction between pre-experimental intelligence beliefs, the manipulation of intelligence mindsets and behavioral self-handicapping. Although in our student sample (N = 101) there was no main effect of the mindset manipulations, participants with more of an initial fixed mindset benefited a lot from a brief growth mindset manipulation and displayed the lowest levels of behavioral self-handicapping. The mindset manipulation had less effect on self-handicapping of originally more of a growth-mindset individuals. These laboratory results demonstrate the benefits of growth mindset triggers which can be especially beneficial to reduce self-handicapping of young adults with more of a fixed mindset in educational settings.

## Introduction

As an educator one may ask what sort of beliefs can promote or undermine their students’ self-handicapping. At school, beliefs about intellectual capacities can become easily triggered by various situations. Students may think that they can alter their intellectual capacities, or they might think that it is something they cannot change. Prior studies demonstrated that if one thinks their intellectual capacities can be improved, it will lead to less maladaptive behaviors such as self-handicapping [e.g.,^1^]. However, in this field, these theoretically reasonable links were mainly supported by correlational results without manipulating students’ beliefs about their intelligence. The present behavioral study aims to fill this gap with both considering students’ chronic intelligence beliefs as well as manipulating students’ mindset beliefs through specific triggers while using a behavioral measure of self-handicapping.

### Self-handicapping in education

Self-worth theory^2,3^ assumes that the goals to achieve the sense of competency, respect and self-acceptance are the most important human needs. In school, this feeling of worthiness frequently depends on academic achievement. If this is the case, students struggle to avoid failure thus seeming to be incompetent, instead they are concerned with appearing able and worthy in front of their peers. Indeed, Covington and Omelich^4^ found among students that reputation of being exceptionally smart was the most important contributor to feelings of personal well-being, far more relevant than their GPA. Among other behaviors, self-handicapping can be a suitable tool to preserve and protect self-esteem in such academic situations^5^. In these cases, students do not exert effort, postpone studying until the last moment or attend in performance-inhibiting activities (e.g., going out before an important performance situation), so subsequent failure may be attributed to non-ability, rather unstable, controllable, and specific causes. Such handicaps, erected by students themselves, allow them to draw attention to other factors than low ability in case of failure, thus minimizing the impact of failure on their self-image and protecting self-esteem. In other words, self-handicapping represents attempts to control attributions about the self and the implications for self-esteem of anticipated or potential failure by establishing a non-ability explanation for its cause^6,7^. Self-handicapping occurs when the individual’s self-concept is under threat, and the uncertainty about one’s ability is high^8^. Likewise, these attributions are beneficial because they support sustaining the motivation and positive emotions in case of either failure or success^9^. Self-handicapping is grounded in attributions of abilities and therefore the perception of the nature of abilities matters. More specifically, they can be considered as stable or as malleable characteristics, and this is where Dweck’s [see^10,11^] mindset theory becomes relevant.

### Intelligence beliefs

Different views about intelligence are thought to have a profound effect on the way people interpret their performance^12,13^. Previous research has demonstrated that people can think about their abilities in different ways^11,14^. Those holding more of an entity theory or fixed mindset believe that skills and abilities are fixed and relatively stable qualities^14^, and their explanations regarding achievement settings also reflect these theories^15^. Accordingly, they likely adopt performance goals focusing on demonstrating their abilities and searching for positive evaluations from others^14^. However, those with more of an incremental theory or growth mindset believe that abilities and skills are malleable or changeable. Individuals who hold more of a growth mindset believe that these characteristics can be improved with exerting effort (also with others’ support and good strategy). The stronger participants were convinced their ability is malleable, the more they adopted learning goals, chose challenging tasks, and employed adaptive strategies to improve their abilities^14^. Research in achievement contexts such as academics^16^, physical education and sport^17,18^ demonstrated that the performer’s adaptive involvement, affect, cognition and performance are guided by these implicit theories of ability. Specifically, in the academic context, implicit theories about intelligence have been widely examined^19,20^. It was found that implicit theories can create distinct meaning systems^13,15^ that can trigger different patterns of response to challenging academic situations and setbacks^14,21^ and ultimately influencing students' learning processes and achievement^16^. However, it is important to mention that intelligence mindsets are not all-or-nothing concepts, but people can be at different parts of the continuum^11^.

### Self-handicapping and intelligence beliefs in education

Given the attributional foundations of self-handicapping, implicit beliefs about intelligence have been identified as a potential predictor of self-handicapping^22,23^. Based on mindset theory, those students who think that their intellectual abilities are rather stable do not invest energy in self-development (since it is not possible) but strive to show their abilities in front of others and put themselves in the most favorable light possible. Beliefs in fixed ability led to a threatening interpretation of failure in terms of “being dumb and remaining dumb”. For this reason, students with more of a fixed mindset are more likely to engage in self-handicapping than those who regard their abilities as rather changeable since these individuals strive to develop and improve rather than protect themselves^24^.

Prior studies found significant relationships between implicit theories of ability and self-handicapping across various domains [e.g.,^1,25^]. For example, an early correlational study^1^ proposed that high trait self-handicapping students are more likely to endorse the entity view of abilities (composite score for athletic ability, intelligence and social skills), than their low trait self-handicapping peers [see also^26,27^]. Likewise, in sports settings, entity beliefs positively associated with self-reported effort reducing and self-reported excuse making, while incremental beliefs negatively associated with self-reported effort reducing^28^. Similar results were found in physical educational contexts, where self-reported inclination to self-handicapping showed similar pattern to implicit ability beliefs^25,29^.

Only a handful of studies investigated such a relationship using behavioral measures. According to Niiya et al.^22^, participants primed with the entity theory practiced less IQ test items (sign of behavioral self-handicapping) than those primed with the incremental theory. Similarly, following a failure experience, participants who had been listening to an entity message about giftedness engaged in behavioral self-handicapping to a greater degree than those who had been exposed to an incremental message about giftedness^23^. In a study of Rickert et al.^30^ the strength of one’s entity theory of intelligence was positively associated with self-reports of academic self-handicapping and was negatively associated with self-reported study time and effort measured by daily records. Additionally, Brown et al.^31^ found that even women self-handicapped behaviorally (who normally stay away from it^36^) after failure when their growth motivation was low.

These previous studies did not examine whether some of the participants are more sensitive to the mindset inductions in terms of their self-handicapping behaviors. One may suppose that meeting a congruent belief (prior growth mindset with a growth mindset induction) would lead to stronger beneficial effect, since an adaptive belief system is being activated and reinforced. Another reasoning could be that students with more of a fixed mindset (similarly to academically at-risk students with lower prior performance^16,32^) would benefit more from the growth mindset induction as there is more room for positive changes in terms of relevant behaviors, such as reducing self-handicapping. We expected that students on the fixed-mindset side of the continuum can benefit more from the growth mindset induction as it provides more room for threat reduction that can result in less self-protective behaviors. However, students on the fixed mindset side of the continuum who receives a fixed mindset induction would experience an enhanced level of threat leading to extreme levels of self-handicapping. On the other hand, we expected that the effect of the induced growth or fixed mindset would be smaller among students on the growth mindset side of the continuum [see also^33–35^].

With an analogy, growth mindset induction can be imagined as a shield against threat. The effect of the shield would be smaller among those who already have an armor (initial growth mindset side) than among those who do not have one and are more exposed to threats (initial fixed mindset side). Giving a growth mindset induction as a shield or enhancing the threat with a fixed mindset induction can be especially important to protect “unarmed selves” (students with strong initial fixed mindset). Therefore, we can expect a larger effect of the inductions on this side of the mindset spectrum in terms of using self-protective behaviors such as self-handicapping.

Since most prior studies examined the link between implicit theory of intelligence and self-handicapping with self-reported methods, the causal link between the two constructs might require further investigations through lab experiments. Furthermore, little is known not only about how experimentally altered mindsets can change self-handicapping, but also about the interactive effects of pre-experimental (chronic) mindsets and the experimentally manipulated mindsets on self-handicapping. More specifically, no prior studies examined whether an initially more of a fixed vs. growth mindset student can profit more from a growth mindset manipulation if we focus on their self-handicapping behaviors.

We expected that students who are on the fixed side of the mindset spectrum will be more sensitive to the growth vs. fixed mindset inductions. Among them the growth mindset induction as an induced self-protection will lead to less self-handicapping behaviors than among students who are already more protected by growth mindset beliefs. On the other hand, we also expected that self-handicapping as a self-protective behavior will be strongly present among those who experience the strongest self-threat: students on the fixed side of the continuum after a fixed mindset induction. We did not expect salient differences among students who are initially on the growth side of the intelligence mindset continuum.

## Methods

### Participants

Similar to earlier laboratory studies^22,23,31^, we aimed to collect data from about 110 students. In the present work, participants were male university students (N = 105), as based on prior studies [e.g.,^36^] males use more behavioral self-handicapping than females. Four participants were dropped from the analyses due to expressing general suspicion of the deception utilized in the study, resulting in a final sample of 101 participants. Statistical analyses were conducted on this final sample. The participants were between 18 and 55 years of age (*M*_age_ = 27.33 years, *SD*_age_ = 8.79 years). Participants received partial course credit for their participation. The study was conducted in accordance with the Declaration of Helsinki and with the approval of the Research Ethics Committee of Eötvös Loránd University, Hungary as well as the informed consents of the participants.

### Procedure

**Figure 1:**
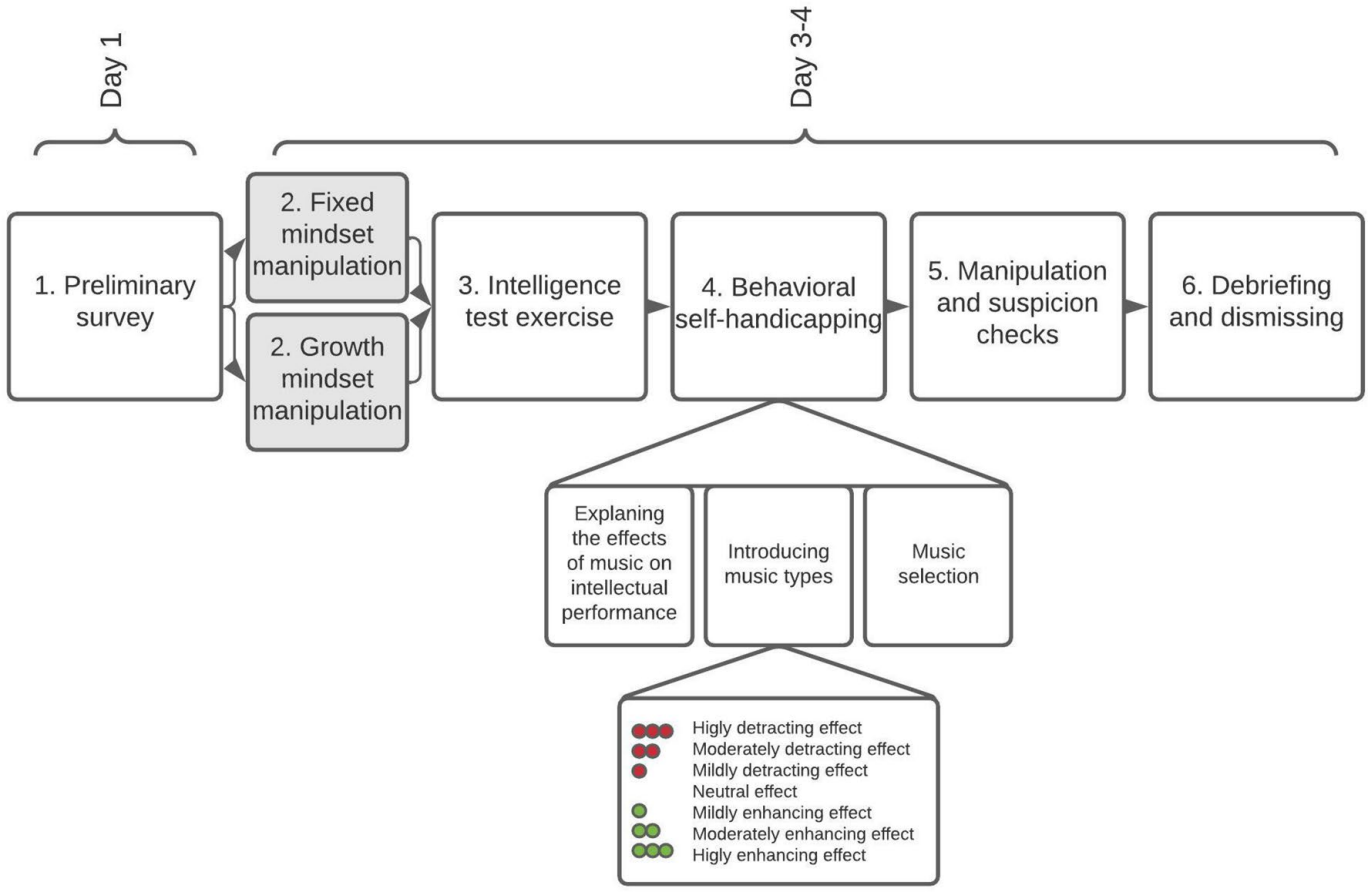
The procedure of the experiment. Participants were invited to participate in a laboratory study—allegedly—in order to validate a test to measure intellectual skills among Hungarian university students. First, participants filled out a preliminary online questionnaire set (e.g., demographic data, personal importance of intelligence, implicit intelligence theory) prior to the laboratory experiment. A few days later, upon arrival to the laboratory they were randomly assigned to one of the two experimental conditions (fixed/growth intelligence mindset). Then they were provided two solvable and four unsolvable “trial” intelligence test items. This induction was supposed to generate failure expectations regarding the following “real” intelligence test. However, before the “real” test, participants were given an opportunity to self-handicap by selecting one out of seven types of music that were characterized by having enhancing, neutral or detracting effect on their test performance. Therefore, music selection represented behavioral self-handicapping. Finally, manipulation and suspicion checks were implemented.

The research procedure is shown in Fig. 1. Participants were told that they can contribute to the validation of an online IQ test based on its reliable offline version.

1. *Preliminary survey*: First, they completed an online survey with items related to their intelligence beliefs and socio-demographic characteristics (see below in details). Two or three days later they were invited to the laboratory to participate in an experiment. The behavioral data were collected individually in a small university laboratory in which participants were randomly assigned to one of the two experimental conditions: (a) entity, (b) incremental mindset.
2. *Manipulation of intelligence mindsets*: Upon arrival at the laboratory participants had to wait for a few minutes in the corridor, where scientific posters illustrated with pictures and diagrams were pinned on the wall either about the heritability of the intellectual skills (entity condition), or the malleability of the brain (incremental condition). Then participants were greeted by the experimenter wearing white lab coat who accompanied them to the laboratory room. Next, the experimenter delivered the implicit message manipulation. In the entity condition, participants were told the following:
  In this research intellectual skills are investigated. As you must know, intelligence is defined by an IQ score. When a baby is born, they already possess a particular intellectual potential that is unfolding throughout their life. This means that our intellectual skills are barely changeable, instead they predominantly remain stable and fixed through the lifespan. Hence, we are not able to do anything to change our intellectual skills. This research (the experimenter points to the same scientific report pinned on the wall that they had seen before) for instance is significant for this reason. Thompson and his colleagues studied identical twins in 2002. Identical twins are known to be 100% identical in terms of their DNA. The authors found that intellectual skills are 88% identical in the two siblings. This means that intellectual skills are highly determined by our genes. This is the reason behind the intelligence not changing throughout our lifespan.
  In the incremental condition, participants were told the following:
  In this research intellectual skills are investigated. Numerous previous studies showed that our intellectual skills change through the lifespan. This process depends on how much we learn and the amount of effort we exert. So, the scientific reports from the last few years evidently showed us that the structure of the brain develops and changes when we work hard on a task or exert great intellectual effort. As a result, new brain connections form and the existing connections strengthen. As such, human brain and intellectual skills can be developed. This research (the experimenter points to the same scientific report pinned on the wall that they had seen before) for instance is significant for this reason. Zatorre and his colleagues studied ordinary people learning to juggle in 2013. They found that after just one week of learning participants’ gray matter density increased, demonstrating development of their brains. This is the reason behind the statement that intellectual skills can be cultivated.
  Both conditions additionally heard some statements about the importance of the intelligence:
  Nonetheless, we all know that intelligence is a crucial attribute of ourselves. Intelligence affects the grades we receive, the university and the job we get into, so basically and ultimately how successful we will be in our lives. Thus, intelligence is really an important factor.
3. *Intelligence test exercise*: Subsequently, participants were told that they would be exposed to some “trial” items before the “real” IQ test in order to assure that they understood the task and were familiar with the type of it. Participants were told that confounding factors such as misunderstanding needs to be ruled out so the real IQ test score would reflect their real intellectual skills. Six items were selected from the Raven Progressive Matrices^37^ and the last four were modified to be unsolvable. Participants had six minutes to complete the tasks and they were informed about the status of the countdown. After the 6th minute the experimenter took the sheet, and visibly flipped through it.
4. *Behavioral measure of self-handicapping*: Then participants were informed that the validation process needs to be explored in various circumstances. One of these circumstances is related to the effect of music on their performance. Therefore, the experimenter asked the participants to choose one of the available CDs to listen to while completing the IQ test^22,31,38^. According to the cover story, the music on each CD has a different effect on their cognitive functions. The CDs were stacked horizontally (so that the colored labels could be easily seen) in a CD rack and labelled: with Three green dots “*Highly Enhancing*” (1), Two green dots “*Moderately Enhancing*” (2), One green dot “*Mildly Enhancing*” (3) without a sign “*Neutral*” (4), One red dot “*Mildly Detracting*” (5), Two red dots “*Moderately Detracting*” (6) and Three red dots “*Highly Detracting*” (7) effects. The experimenter emphasized that the data collection is about to finish so there were enough music choices from all types. Therefore, they are free to choose whatever they really wish to listen to. Participants were left alone for one minute, then handed the selected CD to the experimenter.
5. *Manipulation and suspicion checks*: While the experimenter allegedly started to mount the music device, participants were requested to answer some manipulation and suspicion checks. They were asked about their actual intelligence mindset (two items, see below) as a manipulation check. We also asked participants regarding their CD choice ("*What kind of effect the red/green signed music has on the cognitive functions?*") in order to confirm that participants understood the meaning of selecting a particular type of music^23,31^. The potential suspicious effects were also tested using a funnel method^39^. First, participants were asked, “*How could you describe the purpose of this study in your own words?*”. Then, they were asked whether they had noticed anything particular during the experiment and especially during the practice session.
6. *Debriefing:* Finally, all participants were thoroughly debriefed directly following the study completion and dismissed.

### Measures

#### Pre- and post-manipulation intelligence mindset beliefs

Students’ implicit theories of intelligence were assessed by the Theories of Intelligence Scale^14^. The scale is composed of two four-item subscales of the entity (e.g., “*Your intelligence is something about you that you can’t change very much*”; Cronbach’s *α* = 0.91) and incremental theories (e.g., “*No matter who you are, you can significantly change your intelligence level*”; Cronbach’s *α* = 0.92) resulting in a unidimensional measure (Cronbach’s *α* = 0.93). The two subscales were interrelated (*r* = − 0.73, *p* < 0.001). Respondents indicated their answers on a six-point Likert scale. All eight items were used pre-manipulation, and two seminal items (“*You have a certain amount of intelligence, and you can’t really do much to change it*” and “*You can always substantially change how intelligent you are*”) of the scale were used post-manipulation as manipulation checks (*r*_inter-item_ = 0.68; *p* < 0.001). The items on the pre- and post-treatment measures were averaged (separately) such that higher values correspond to more growth mindsets.

#### Importance of intelligence

Participants were asked about the importance of the domain that was about to be threatened: “*How important is the level of your intelligence to you?*”. The statement was rated on a 4-point Likert scale from *not at all* (1) to *very much* (5).

#### Behavioral self-handicapping measure

Self-handicapping behavior was operationalized as choosing suboptimal circumstances for performing the target intelligence task. Therefore, prior to taking the second test of intellectual ability, each participant had the opportunity to select one sort of music they wanted to listen to during the IQ test. The music selection ranged on a scale with values from 1 (*highly enhancing*) to 7 (*highly detracting*). The middle of the scale was represented by neutral music. The CD choice constituted the self-handicapping behavior. Based on prior literature we used it as a continuous measure as not choosing the most optimal condition (1-*highly enhancing*) can be interpreted as self-handicapping.

#### Demographic data

Finally, demographic characteristics of participants were asked: gender, age, and whether their intellectual skills had been measured before.

### Statistical analyses

Statistical analyses and data visualization were performed with IBM SPSS software version 22.0 and R 4.0.3^40^. Within R, the tidyverse^41^ and the ggplot2^42^ packages were used for data transformation and visualization. First, we checked potential differences between the control and experimental groups in terms of age, prior intelligence mindset, importance of intelligence, prior participation in intelligence testing and found no differences between the two groups (all *p*s > 0.58). Second, we treated the CD choice behavioral self-handicapping variable as a continuous measure, ordinary least squares (OLS) regression analysis was performed to examine the effect of the experimental manipulation (fixed vs. growth mindset) as well as the interaction between the experimental manipulation and the prior intelligence mindsets. The pre-experimental growth intelligence mindset, the intelligence mindset manipulation and their interaction were included in the regression model. We also estimated the model about the incidence of self-handicapping at − 1 *SD* (towards the fixed direction) and + 1 *SD* (towards the growth direction) of pre-experimental mindsets.

## Results

### Manipulation and suspicion checks

All 101 participants responded correctly to the question about the music selection confirming that they understood the meaning of the CD color signs. For the participants, the level of intelligence was an important personal characteristic (the lowest score was 3 on a 5-point scale, *M* = 3.65, *SD* = 0.48). We also found a significant difference between the two conditions in terms of their post-manipulation growth intelligence mindsets while controlling for prior intelligence beliefs, *b* = 1.63, *t*(2,98) = 9.60, *p* < 0.001, *d* = 1.31.

### The effect of (pre-manipulation and manipulated) intelligence mindset on behavioral self-handicapping

Our first research question was related to the main effect of the fixed vs. growth mindset manipulation on self-handicapping behavior. There was no significant difference, *t*(99) = 1.44, *p* = 0.15, between participants in the growth mindset (*M* = 3.04, *SD* = 2.27) and in the fixed mindset conditions (*M* = 3.70, *SD* = 2.34) in terms of their self-handicapping behaviors. The interaction between pre-experimental intelligence mindset and intelligence mindset manipulation was significant concerning self-handicapping behavior, *b* = 1.21, *t*(3,97) = 2.709, *p* = 0.008, *d* = 0.52.

**Figure 2:**
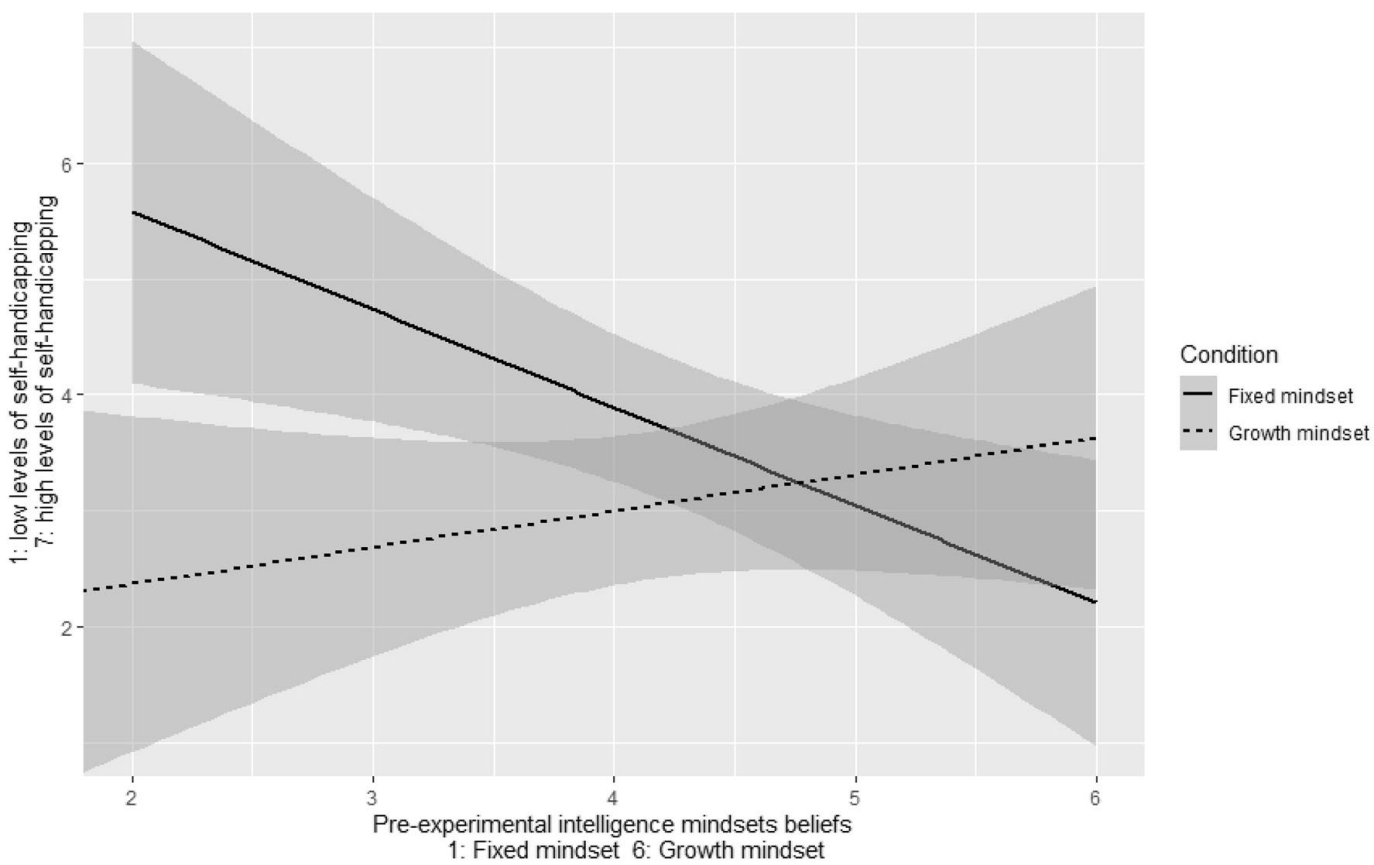
The interaction between prior growth intelligence mindset and intelligence mindset manipulation. The results show that a lower level of prior growth intelligence mindset made participants more vulnerable to behavioral self-handicapping, especially if they met fixed messages of intelligence. Gray shading area represents standard errors.

As displayed on Fig. 2, the experimental manipulation made a significant difference among people with an initially fixed mindset. Participants with stronger pre-experimental fixed intelligence mindset and participating in the fixed mindset condition performed the highest level of self-handicapping. However, participants with similar pre-experimental fixed mindset in the incremental experimental condition displayed much less self-handicapping behavior. The experimental manipulations made a less salient difference among students with more of a pre-experimental growth intelligence mindset.

Tests at ± 1 *SD* of pre-experimental mindsets showed self-handicapping of − 1.90 music selection levels (on a range of 1 to 7), *t*(3,97) = − 3.009, *p* = 0.003, *d* = 0.82, for those who were at − 1 *SD* of prior intelligence mindset (towards the fixed direction), and no effect among those at + 1 *SD* of prior intelligence mindset (towards the growth direction), *b* = 0.52 music selection level on the seven-point measure, *t*(3,97) = 0.831, *p* = 0.41, *d* = 0.23.

In sum, the self-handicapping difference between the mindset inductions appeared clearly among the students who were on the fixed side of the mindset continuum. However, while it resulted in a non-significant increase of self-handicapping among individuals holding more of a growth mindset at baseline, it had an almost four times larger, strongly significant and benevolent effect on the students with more of a fixed mindset at baseline.

## Discussion

According to prior literature, only one experiment examined how manipulated intelligence mindsets can influence behavioral self-handicapping^22^ and found that manipulated fixed mindset leads to elevated self-handicapping (in contrast to manipulated growth mindset). Furthermore, two experiments examined the role of pre-existing mindsets on self-handicapping^23,30^ showing that fixed mindset is associated with an elevated level of self-handicapping. The present study aimed to consider both pre-experimental and manipulated intelligence mindsets in self-handicapping. In this study, we were able to replicate existing findings, but also to extend them to demonstrate how these associations are influenced by prior intelligence mindsets.

We were interested in who might profit more from a growth mindset induction: those who have prior congruent beliefs (pre-experimental growth mindset) or those who “need” it more as they have incongruent pre-experimental fixed mindset beliefs. Based on the results, the second hypothesis is confirmed. It appears that students with more of a fixed mindset at baseline could benefit more from the growth mindset manipulation in terms of reduced self-handicapping (in contrast to students endorsing more of a growth mindset at baseline).

More specifically, students who reported initially more of a fixed mindset and received an experimental growth mindset induction chose less distracting music than students with initially more fixed beliefs in the fixed mindset-promoting experimental condition. The difference among students holding more of a growth mindset after the experimental manipulation was visibly less salient. These results are in line with the above-mentioned studies^22,23,30^ in which growth mindset appears as a protecting shield against self-handicapping. The present results not only confirm these results, but also show that even a very brief mindset induction that took only a few minutes can make a salient difference in the self-handicapping behavior of the vulnerable students with more of a fixed mindset, while such manipulation had less impact on those students who are protected by higher level of prior growth mindset. Additionally, these results are in line with assumptions of Schwinger et al.^43^ showing the effectiveness of even a very brief emotional-motivational manipulation that could reduce the level of self-handicapping.

Self-handicapping can serve as a good excuse in situations with a high risk of failure. However, the need to have and use this excuse can be related to the perceived stakes of the situation that are different for more of fixed vs. growth mindset students. For students being on the fixed mindset side of the continuum, the stakes are high as the threat is related to be seen and to see themselves as “dumb” in an essential, unchangeable manner; therefore, they have a strong affordance to use self-handicapping. However, the stakes and the related threat are smaller for the students with more of a growth mindset (for whom failing is part of the learning process) leading to less self-handicapping^24^. Therefore, our results show that the beneficial effects of growth intelligence mindset supplementation can be especially strong for students with high fixed intelligence beliefs in terms of reducing the level of self-handicapping among men. This result is also in line with previous works, showing that participants with more of a fixed mindset at baseline gained the most from learning an incremental theory in terms of their depressive symptoms^35^, or grades^33,34^. We also showed that, prior growth intelligence beliefs served as a shield against behavioral self-handicapping that may be the consequence of perceiving threat differently (compared to students with highly fixed mindset beliefs). Therefore, our results not just confirm previous research but broaden the understanding of the vulnerability of men with a particular chronic belief system meeting with congruent as well as incongruent messages in an achievement setting.

The present study is not without limitations. First, the procedure was tailored to in-person laboratory situations that can allow identifying causal relationships. However, unfortunately, the pandemic did not enable us to collect a larger sample size. Although our sample size was similar to ones in earlier experimental studies^22,23^, the current results still appear to be somewhat underpowered. The post-hoc power calculation for the interaction term (f^2^ = 0.07, significance level = 0.05 with 3 predictors) showed that the achieved power is slightly below the optimum (0.75). Second, our purpose was to mimic a believable, behavior-based, threatening situation with high ecological validity in which there is an offline performance embedded in an interaction with the experimenter. However, the presence of a white-coated experimenter that created such experimental effects (instead of a unitary online experience) might have led to some inconsistency between participants. Third, behavioral self-handicapping has been conceptualized as choosing a suboptimal context that can reduce their performance [e.g.,^22^] thus other manifestations of behavioral self-handicapping were not investigated. It is important to state that the present one is a preliminary study. Building on its weaknesses, future studies should consider the above-mentioned limitations when investigating experiments or programs that aim to reduce self-handicapping with changing mindset beliefs. For example, as successful intervention programs are available, future studies might investigate the role of growth mindset interventions [e.g.,^16^] in long term changes in self-handicapping behaviors.

## Data Availability

The datasets generated and/or analyzed during the current study are available from the corresponding author on request.
